# Extracellular matrix protein 1 (*ECM1*) is a potential biomarker in B cell acute lymphoblastic leukemia

**DOI:** 10.1007/s10238-023-01255-2

**Published:** 2024-03-28

**Authors:** Li-Xin Wu, Ming-Yue Zhao, Nan Yan, Ya-Lan Zhou, Lei-Ming Cao, Ya-Zhen Qin, Qian Jiang, Lan-Ping Xu, Xiao-Hui Zhang, Xiao-Jun Huang, Hao Jiang, Guo-Rui Ruan

**Affiliations:** 1 Peking University People’s Hospital, Peking University Institute of Hematology, National Clinical Research Center for Hematologic Disease, Beijing Key Laboratory of Hematopoietic Stem Cell Transplantation, Peking University, Beijing, China; 2 Department of Hematology, Peking University Shenzhen Hospital, Shenzhen Peking University-The Hong Kong University of Science and Technology Medical Center, Shenzhen, Guangdong province, China; 3 Peking-Tsinghua Center for Life Sciences, Academy for Advanced Interdisciplinary Studies, Peking University, Beijing, China

**Keywords:** B cell acute lymphoblastic leukemia, *ECM1*, Diagnosis, MRD, Prognosis

## Abstract

B cell acute lymphoblastic leukemia (ALL) is characterized by the highly heterogeneity of pathogenic genetic background, and there are still approximately 30–40% of patients without clear molecular markers. To identify the dysregulated genes in B cell ALL, we screened 30 newly diagnosed B cell ALL patients and 10 donors by gene expression profiling chip. We found that *ECM1* transcription level was abnormally elevated in newly diagnosed B cell ALL and further verified in another 267 cases compared with donors (median, 124.57% vs*.* 7.14%, *P* < 0.001). ROC analysis showed that the area under the curve of *ECM1* transcription level at diagnosis was 0.89 (*P* < 0.001). Patients with *BCR::ABL1* and *IKZF1* deletion show highest transcription level (210.78%) compared with *KMT2A* rearrangement (39.48%) and *TCF3::PBX1* rearrangement ones (30.02%) (all *P* < 0.05). Also, the transcription level of *ECM1* was highly correlated with the clinical course, as 20 consecutive follow-up cases indicated. The 5-year OS of patients (non-*KMT2A* and non-*TCF3::PBX1* rearrangement) with high *ECM1* transcription level was significantly worse than the lower ones (18.7% vs*.* 72.9%, *P* < 0.001) and high *ECM1* transcription level was an independent risk factor for OS (HR = 5.77 [1.75–19.06], *P* = 0.004). After considering transplantation, high *ECM1* transcription level was not an independent risk factor, although OS was still poor (low vs*.* high, 71.1% vs*.* 56.8%, *P* = 0.038). Our findings suggested that *ECM1* may be a potential molecular marker for diagnosis, minimal residual disease (MRD) monitoring, and prognosis prediction of B cell ALL.

*Trial registration* Trial Registration Registered in the Beijing Municipal Health Bureau Registration N 2007–1007 and in the Chinese Clinical Trial Registry [ChiCTR-OCH-10000940 and ChiCTR-OPC-14005546]; http://www.chictr.org.cn.

**Supplementary Information:**

The online version contains supplementary material available at 10.1007/s10238-023-01255-2.

## Introduction

B cell acute lymphoblastic leukemia (B cell ALL) is a malignant clonal disease originating from lymphoid hematopoietic stem cells. It presents highly heterogeneous clinical manifestation, which is fundamentally the heterogeneity of pathogenic genetic background [1]. According to these studies, the heterogeneity may mainly attribute to the following two aspects: (1) diverse recurrent somatic alterations. These alterations encompass aneuploidy, chromosomal rearrangements, DNA copy number alterations, and sequence mutations, referred to as primary genetic abnormalities to define distinct subtypes [2]. Recently, Gu et al*.* [3] performed RNA sequencing in B cell ALL and identify several novel driver events with different gene expression signature, subdividing the subtypes to as many as 23. (2) Cooperating or secondary aberrations. Studd et al*.* [4] found that additional mutation burden varied significantly across subtypes. Patients with intrachromosomal amplification of chromosome 21 (iAMP21) harbored the highest burden, and the *KMT2A* rearrangement (*KMT2A*-r) ones the lowest [4]. Besides, under the selection pressure of treatment, additional aberrations would be lost, gained or enriched, termed as clonal evolution [5].

Advances in genetic testing methods have made it possible for us to fully understand the molecular regulatory network in B cell ALL. Over the past decade, genomic analysis has revolutionized the cognition of researchers and clinicians on the biology of B cell ALL [6]. For example, patients with high hyperdiploid ALL generally up-regulate gene expression on the gained chromosomes in dosage effect. However, Yang et al. found that *CTCF*, which regulates chromatin architecture, displayed low expression in these patients [7]. Another important notion in B cell ALL is “phenocopy,” which refers to the cases with alternative or unknown driver events show similar gene expression profile to the well characterized drivers [8]. The best known is Ph-like ALL, which copies the signature of ones with *BCR::ABL1* [9]. Recently, more and more phenocopies were identified, including *ETV6::RUNX1*-like, *KMT2A* rearrangement-like, hyperdiploid like and so on [4, 10, 11]. These findings improve the stratification of B cell ALL and offer potential treatment strategies for these newly defined subgroups.

Here, to further unveil the aberrations in B cell ALL, we used gene expression profiling chip in 30 newly diagnosed B cell ALL patients and 10 donors. We found that extracellular matrix protein 1 (*ECM1*) was up-regulated in B cell ALL. Our data suggested that *ECM1* was a potential biomarker for diagnosis, minimal residual disease (MRD) monitoring and prognosis prediction of B cell ALL.

## Methods

### Patients and treatments

In this study, we collected bone marrow samples and corresponding clinical data from newly diagnosed (*N* = 297), complete remission (CR, *N* = 41), relapsed (*N* = 42) B cell ALL patients and healthy donors (*N* = 32), from March 2012 to December 2018 in Peking University People's Hospital. The diagnostic criteria for B cell ALL refer to the 2016 WHO guidelines. All patients received standard chemotherapy regimens. According to physician evaluation and patient conditions, consolidation therapy could be chemotherapy or allogeneic hematopoietic stem cell transplantation. Among them, *BCR::ABL1* positive patients were treated with tyrosine kinase inhibitors. This study was approved by the Ethics Committee of Peking University People's Hospital in accordance with the Declaration of Helsinki. All patients signed informed consent.

### Immunophenotype, MRD and cytogenetic analyses

Multi-parameter flow cytometry (MFC) was used to identified the immunophenotype at diagnosis. The antibody combinations could be referred to the previous report [12]. MRD was assessed by MFC and molecular level. For MFC, leukemia-associated immunophenotypes were monitored. The sensitivity is 10^–4^ and level ≥ 10^–4^ was considered MFC-MRD positivity. For molecular level, fusion genes (*BCR::ABL1*, *KMT2A*-r, *TCF3::PBX1*, *ETV6::RUNX1*) and *IKZF1* deletion were monitored if ones harbored any above-aberration. Transcription level of *WT1* was routinely monitored and > 0.60% was defined as positive. Cytogenetic analysis was done by standard G-banding and/or FISH [13].

### Gene expression microarray

Mononuclear cells were isolated from bone marrow samples by Ficoll-Hypaque density gradient centrifugation (Solarbio Technology, Beijing, China). RNA was extracted by Trizol^®^ kits (Invitrogen, Carlsbad, CA, USA) according to the manufacturer’s instructions. A total of > 1 ug RNA was used from each of the 30 newly diagnosed B cell ALL patients and 10 donors for gene expression microarray.

### Reverse transcription and quantitative real-time polymerase chain reaction (qRT-PCR)

Reverse transcription was performed as previously described [14]. Taqman-based qRT-PCR was performed by ABI PRISM^®^ 7500 Fast Sequence Detection System (Applied Biosystem, Foster City, CA, USA) [15]. *ABL1* was used as internal control. Primers and probes were shown as follows: *ECM1*, forward primer, 5′-CAAAGAGTTCTCACCAAGCATAAACA-3′; reverse primer, 5′-TCTGGAAATGGCAGGTCACA-3′; probe, 5′-FAM-TGGGCTGATCCACAACATGACTGCC-BHQ-3′. *ABL1*, forward primer, 5′-TGGAGATAACACTCTAAGCATAACTAAAGGT-3′; reverse primer, 5′-GATGTAGTTGCTTGGGACCCA-3′; probe, 5′-FAM-CCATTTTTGGTTTGGGCTTCACACCATT-TAMRA-3′.

### GEO dataset validation

B cell ALL dataset GSE34861 was retrieved from GEO database to analyze the basic information and prognosis of patients with different transcription level of *ECM1*, further verifying the clinical significance of *ECM1* in B cell ALL.

### Statistical analysis

Statistical analysis was performed using SPSS 22.0 and Graphpad Prism 7.0. Continuous variables were represented by median and full range and compared by Mann–Whitney U test. Categorized variables were compared by chi-square test or Fisher′s exact test. Correlation of two variables was analyzed by Spearman correlation test. The receiver operator characteristic (ROC) curve was used to evaluate the diagnostic sensitivity and specificity of *ECM1*. Overall survival (OS) is defined as the patient′s survival time from initial diagnosis to death from any cause, last contact, or December 31st, 2020. Kaplan–Meier method was used to estimate OS and log rank test was performed to compare. Variables with *P* < 0.1 were enrolled in the multivariate analysis using a Cox proportional risk regression model. *P* < 0.05 is considered statistically significant.

## Results

### Dysregulated gene expression in B cell ALL

A total of 30 newly diagnosed B cell ALL and 10 normal donors were enrolled for gene expression microarray. Patient characteristics are shown in Supplementary Table 1. There were 19 (63%) males, and the median age was 33 years old. Most of them were common-B ALL (*N* = 22, 73%), and the most common cytogenetics abnormality is t (9;22)(q34;q11). Correspondingly, *BCR::ABL1* fusion accounted for nearly half of the patients (*N* = 14, 47%). *IKZF1* deletion (*N* = 14, 47%) often concurred with *BCR::ABL1*, and 8 ones both harbored *BCR::ABL1* fusion and *IKZF1* deletion (double-positive). There were 10 double-negative cases, while *IKZF1*-positive and *BCR::ABL1*-positive were 6 cases each. Five cases had *KMT2A*-r fusion, one had *TCF3::PBX1* fusion and none of them had *ETV6::RUNX1* fusion. As for donors, there were 6 males and the median age was 43 years old.

We firstly subdivided B cell ALL patients into 4 subgroups according to the *BCR::ABL1* and *IKZF1* status to unveiled the dysregulated genes in different background. As shown in Supplementary Fig. 1 and Supplementary Table 2, the most significant difference was seen in double-positive and double-negative group. In the double-negative group, the up-regulated genes were 538 more than that in the double-positive group, but the down-regulated genes were significantly reduced, reaching 1744.

### ECM1 had high transcription level in B cell ALL

**Fig. 1:**
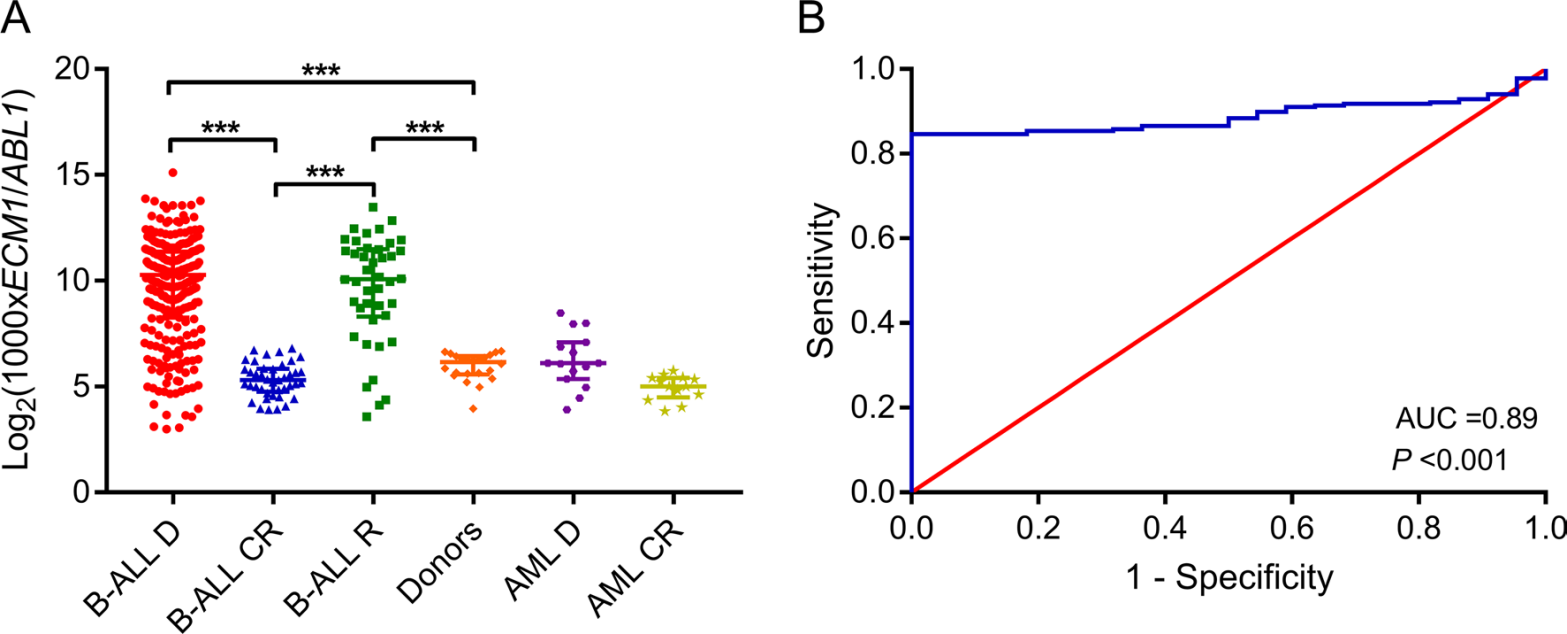
Up-regulated transcription level of *ECM1* in newly diagnosed B cell ALL. **A** Transcription level of *ECM1* in B cell ALL (*D* newly diagnosed, *CR* complete remission, *R* relapse), normal donors, and AML (*D* newly diagnosed, *CR* complete remission). The original transcription level (*ECM1*/*ABL1*) was multiplied by 1000 times and then logarithmically conversed. **B** The ROC curve of *ECM1* transcription levels for B cell ALL diagnosis

We found that the two transcripts (NM_004425, NM_022664) of *ECM1* were both highly transcribed in the four subgroups (Supplementary Table 3), indicating that the abnormal expression of *ECM1* may have universal significance in B cell ALL. To elucidate the possibility of *ECM1* as a potential molecular marker in B cell ALL, we further verified the transcription level of *ECM1* in samples from newly diagnosed (*N* = 267), complete remission (CR, *N* = 41) and relapsed (*N* = 42) B cell ALL, as well as 22 healthy donors. Basic clinical information of 267 newly diagnosed patients was shown in Supplementary Table 4. Particularly, most of the patients (97%, 259/267) were adults (age ≥ 14 years), and only 8 patients aged < 14 years. Our data showed that the highest level of *ECM1* transcription was found in newly diagnosed B cell ALL patients, with a median of 124.57% (0.79–3519.50%), significantly higher than in CR patients (median, 3.95% [1.50–11.22%], *P* < 0.001) and donors (median, 7.14% [1.54–10.27%], *P* < 0.001). Transcription level elevated again in relapsed patients, with a median of 107.62% (1.19–1142.81%), and was significantly higher than in CR patients (*P* < 0.001) and donors (*P* < 0.001) (Fig. 1A). We also detected *ECM1* transcription level in AML, including 15 newly diagnosed and 13 CR samples. The median transcription level of newly diagnosed AML was 6.88% (1.50–35.76%), and the median in CR was 3.21% (1.43–5.36%). There was no significant difference in *ECM1* transcription levels between newly diagnosed AML and normal donors (*P* = 0.551). ROC analysis of *ECM1* transcription level in newly diagnosed B cell ALL patients showed that the area under the curve was 0.89 (95% confidence interval, 0.85–0.93; *P* < 0.001) (Fig. 1B). The maximum Youden index is 0.846, corresponding to an *ECM1* transcription level of 10.28%. Taking 10.28% as the diagnostic threshold, the sensitivity and specificity for diagnosis was 84.6 and 100%, respectively. We further compared the diagnostic performance of *ECM1* with *WT1* in B cell ALL. In 265 patients who were tested for both *ECM1* and *WT1*, the overall false negative rate of *ECM1* in diagnosing B cell ALL was 15.1%, while the false negative rate of *WT1* was 34.3%. The overall false negative rate of the two genes was 4.2% (Supplementary Table 5). Therefore, we suggested that *ECM1* is more sensitive than *WT1* in diagnosing B cell ALL, and these two genes could complement each other.

**Fig. 2:**
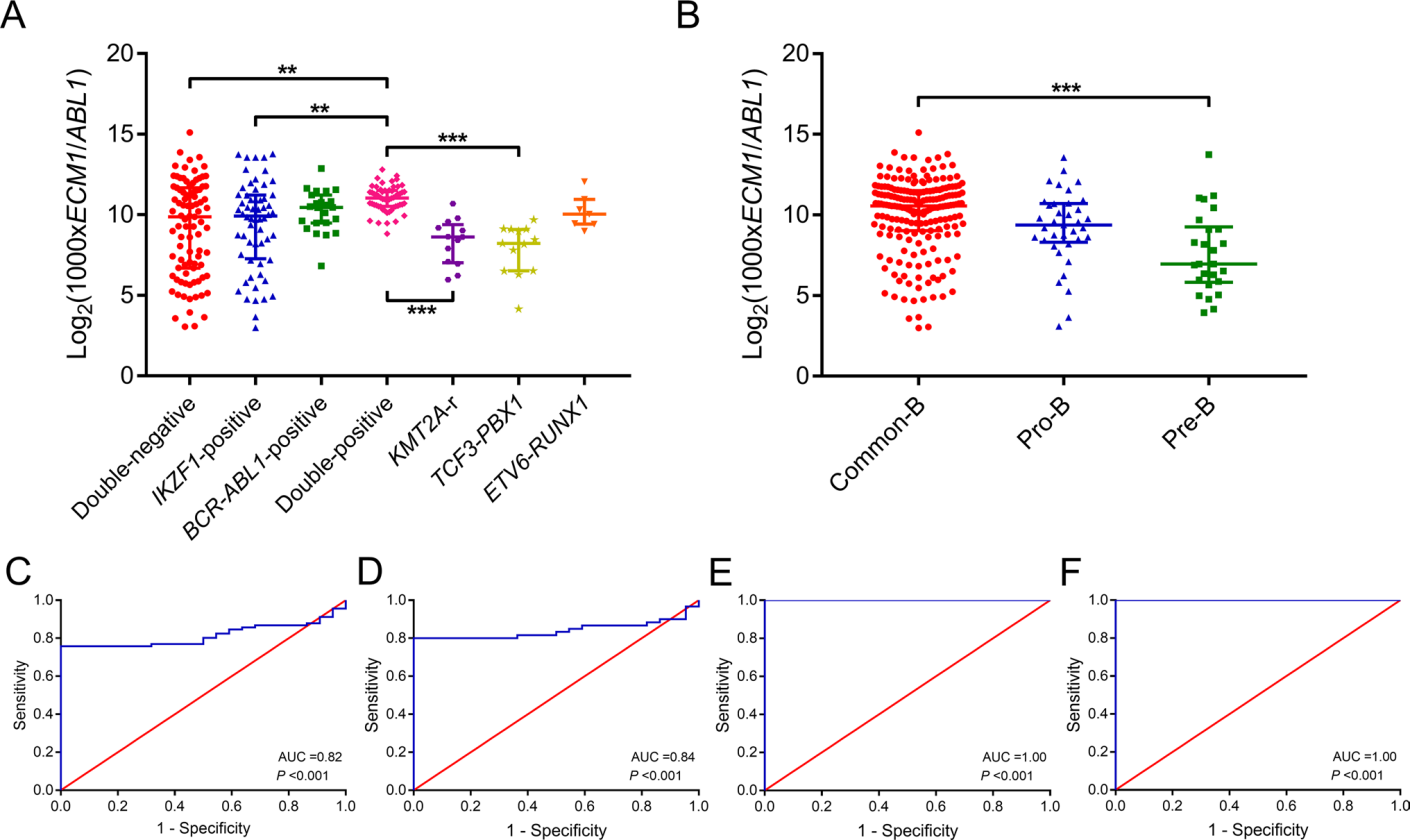
Transcription levels of *ECM1* in different B cell ALL subgroups. **A** Transcription levels of *ECM1* in different genetic backgrounds. **B** Transcription levels of *ECM1* in different immunophenotypes. The original transcription level (*ECM1*/*ABL1*) was multiplied by 1000 times and then logarithmically conversed. **C**–**F** The ROC curve of *ECM1* transcription level in the double-negative, *IKZF1*-positive, *BCR:ABL1*-positive, and double-positive groups, respectively. **: *P* < 0.01, ***: *P* < 0.001

We also compared the transcription levels of *ECM1* among different subgroups (Fig. 2A–B). Among them, patients with *KMT2A*-r, *TCF3::PBX1*, and *ETV6::RUNX1* were further grouped independently. Multiple comparison showed that there was no significant difference in transcription levels among the double-negative (median, 93.50% [0.83 – 3519.50%]), *IKZF1*-positive (median, 97.77% [0.79 – 1397.76%]), and the *BCR::ABL1*-positive subgroups (median, 141.22% [11.33 – 754.35%]) (both *P* > 0.05). Double-positive group showed the highest transcription level, with a median level of 210.78% (45.30 – 714.05%). Patients with *KMT2A*-r and *TCF3::PBX1* had the lowest transcription levels, with a median of 39.48% (6.28 – 165.32%) and 30.02% (1.78 – 82.54%), respectively. However, the transcription level of patients with *ETV6::RUNX1* was not low (median, 104.87% [50.84 – 424.05%]). The transcription level in the double-positive ones was significantly higher than that in the double-negative (*P* = 0.001), the *IKZF1*-positive (*P* = 0.005), *KMT2A*-r (*P* < 0.001), and *TCF3::PBX1* ones (*P* < 0.001). As for immunophenotypes, Common-B ALL had the highest transcription level (median, 151.53% [0.79–3519.50%]), significantly higher than that in the Pre-B ALL (median, 12.45% [1.54–1373.54%]; *P* < 0.001). Transcription level in Pro-B ALL was also not high (median, 66.48% [0.85–1210.48%]; *P* = 0.053). We further investigated the diagnostic performance of *ECM1* in patients except for *KMT2A*-r, *TCF3::PBX1*, and *ETV6::RUNX1* rearrangements (Fig. 2C–F). In the double-negative group, the area under the curve was 0.82 (0.74–0.89; *P* < 0.001), slightly lower than that in the *IKZF1*-positive group (0.84 [0.76–0.93]; *P* < 0.001). However, in *BCR::ABL1*-positive and double-positive groups, the area under the curve was 1 (*P* < 0.001).

### ECM1 had a good MRD monitoring performance

**Fig. 3:**
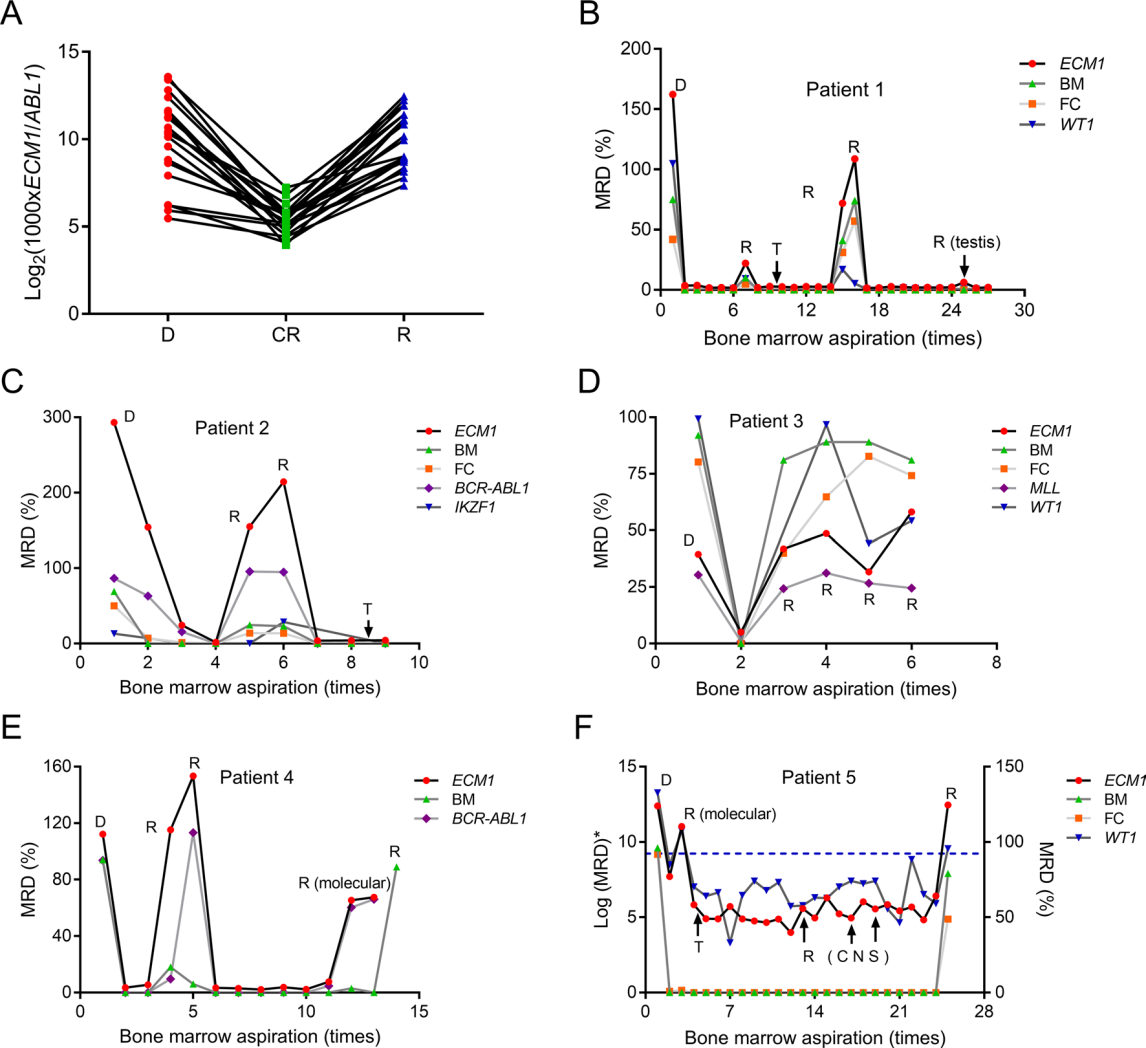
MRD monitored by different indicators. **A** The correlation between *ECM1* transcription level and clinical course in consecutive 20 patients. The original transcription level (*ECM1/ABL1*) was multiplied by 1000 times and then logarithmically conversed. **B**–**F** Transcription level of *ECM1* and other clinical MRD monitoring indicators in 5 long-term follow-up patients. Samples at diagnosis, relapse, molecular relapse, and extramedullary relapse were marked, while the remaining unlabeled points indicated complete remission. The actual MRD levels of each indicator were shown in patients 1–4. Due to the significant difference in transcription levels between *ECM1* and *WT1* in patient 5, *ECM1* was multiplied by 1000 times and then logarithmically conversed, while *WT1* was multiplied by 10,000 times and then logarithmically conversed, corresponding to the left axis. The blue dashed line represented to the *WT1* threshold (0.60%). Bone marrow morphology and flow cytometry were showed as the actual MRD levels, corresponding to the right axis. *D* diagnosis, *CR* complete remission, *R* relapse, *T* transplantation, *CNS* central nerve system, *BM* bone marrow morphology, *FC* flow cytometry

We further evaluate the value of *ECM1* transcription level for MRD monitoring. In 20 consecutive follow-up cases, it was found that *ECM1* transcription level down-regulated after achieving CR, but up-regulated at the time of relapse (Fig. 3A). We further monitored MRD of 5 long-term follow-up cases and compared *ECM1* detection with other MRD monitoring techniques (morphology, flow cytometry, other genes in qRT-PCR) (Fig. 3B–F). All patients showed high *ECM1* transcription level at diagnosis. Patients with long-term CR also maintained low transcription level of *ECM1* (patients 1, 4, and 5), while refractory or relapse patients maintained high transcription level (patient 3). Relapsed patients after transplantation also showed elevated *ECM1* transcription level (patients 1, 5).

As for morphology, the *ECM1* transcription level was basically consistent with the blast at most bone marrow aspirations. In some patients, *ECM1* transcription level detection was superior to morphology. For patient 2, after induction (the 2nd bone marrow aspiration), the morphology showed CR, but MFC and genes (*BCR::ABL1*, *IKZF1*) did not achieve, as well as *ECM1* transcription level. Patient 4 relapsed during the 4th and 5th aspirations, and the sensitivity of *ECM1* was superior to morphology. Molecular relapse occurred during the 12th and 13th aspirations, with an increase in *ECM1* transcription level, but morphological relapse was at the 14th aspiration. Patients 1, 2, 3, and 5 provided follow-up MFC information. In patients with MFC-MRD, the transcription level of *ECM1* was highly consistent with the MFC, especially in the relapsed samples. In terms of other genes, the transcription level of *BCR::ABL1* (patients 2 and 4) and *KMT2A*-r (patient 3) showed good parallelism with *ECM1* in disease course monitoring. Patient 2 also had *IKZF1* deletion. The dynamics of *IKZF1* deletion level and *ECM1* transcription level was basically consistent. Patients 1, 3, and 5 provided *WT1* follow-up information. The dynamics of *WT1* transcription level was basically consistent with *ECM1*. Like other intramedullary MRD indicators, due to the fact that two patients with extramedullary recurrence (patients 1 and 5) did not suffer intramedullary relapse simultaneously, the transcription level of *ECM1* did not indicate the status of extramedullary relapse well.

### High ECM1 transcription level indicated worse OS

We divided 177 adult (age ≥ 14 years) B cell ALL patients with prognostic information into a high transcription level group (*N* = 86) and a low transcription level group (*N* = 91) based on the median transcription level of *ECM1* (124.57%) (Table 1). In the high transcription level group, patients presented high-risk clinical features, such as older age (high transcription level group vs*.* low transcription level group, 35 y vs*.* 29 y, *P* = 0.008), high positive rate of MFC-MRD (≥ 0.01%) after induction (7 patients without MFC-MRD information) (64% vs*.* 43%, *P* = 0.005), high positive rate of MFC-MRD after 1 course of consolidation chemotherapy (18 patients without MFC-MRD information) (46% vs*.* 27%, *P* = 0.010) and after 2 courses of consolidation chemotherapy (33 patients without MFC-MRD information) (43% vs*.* 22%, *P* = 0.008). In terms of immunophenotype, patients with high transcription level were more likely Common-B ALL (*P* = 0.001). This result was consistent with the molecular genetics classification, because *KMT2A*-r is more common in the Pro-B ALL, while *TCF3::PBX1* rearrangement is more common in the Pre-B ALL. The distribution of *BCR::ABL1* and *IKZF1* between the high and low transcription level groups was consistent with the above results. Patients with high transcription level were more likely to have *BCR::ABL1* and *IKZF1* deletion mutations (*P* < 0.05). There was no significant difference between the two groups in terms of gender, white blood cell count, hemoglobin concentration, platelet count, *ETV6::RUNX1* rearrangement, and treatment regimen (all *P* > 0.05).

**Table 1:** Characteristics of 177 B cell ALL patients

Variables	*ECM1* high (*N* = 86)	*ECM1* low (*N* = 91)	*P*-value
Sex, *N* (%)			0.159
Male	45 (52)	38 (42)	
Female	41 (48)	53 (58)	
Age, y			
Median (range)	35 (14–71)	29 (14–67)	0.008
WBC, × 10^9^/L			
Median (range)	17.1 (0.8–337.6)	14.1 (1.0–358.0)	0.896
Hemoglobin, g/L			
Median (range)	89 (35–164)	92 (35–157)	0.568
Platelets, × 10^9^/L			
Median (range)	44 (3–377)	57 (3–352)	0.148
Immunophenotype, *N* (%)			0.001
Common-B	73 (85)	60 (66)	
Pro-B	9 (10)	18 (20)	
Pre-B	2 (2)	13 (14)	
Unknown	2 (2)	0	
Molecular aberrations, *N* (%)			
* BCR::ABL1*	39 (45)	15 (16)	< 0.001
* IKZF1* deletion	54 (63)	34 (37)	0.001
* KMT2A*-r	0	8 (9)	0.007
* TCF3::PBX1*	0	9 (10)	0.003
* ETV6::RUNX1*	1 (1)	2 (2)	1.000
MRD ≥ 0.01%, *N* (%)			
Induction	52 (64)	38 (43)	0.005
1st course consolidation	37 (46)	21 (27)	0.010
2nd course consolidation	31 (43)	16 (22)	0.008
Treatment, *N* (%)			
Transplantation	60 (70)	70 (77)	0.281

**Fig. 4:**
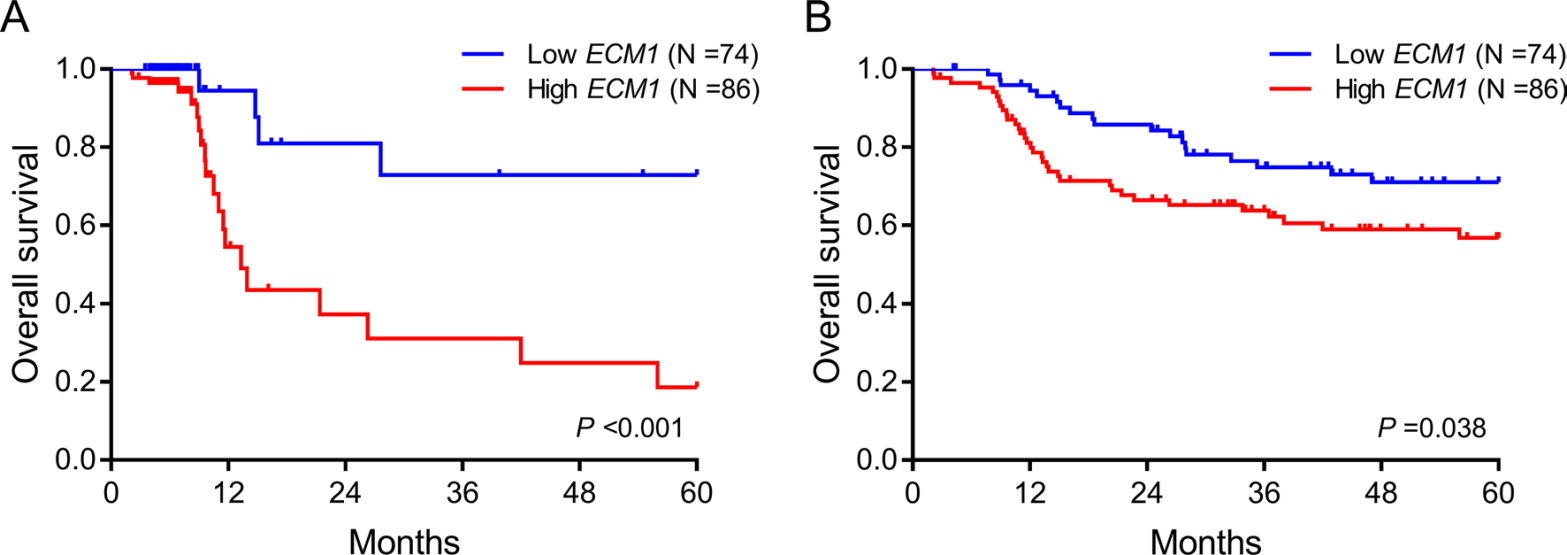
Prognostic analysis of *ECM1* in B cell ALL patients. **A** OS analysis with transplantation as censored event. **B** OS analysis with transplantation not as censored event

The specific decreased *ECM1* transcription level in *KMT2A*-r (*N* = 8) and *TCF3::PBX1* (*N* = 9) patients suggested that the mechanism of *ECM1* in these two subtypes may be different from that of non-*KMT2A*-r and non-*TCF3::PBX1* patients. So, subsequent prognostic analysis did not include these patients. In the case of transplantation as censored event, the 5-year OS of patients with high *ECM1* transcription level was significantly lower than patients with low transcription level (low vs*.* high, 72.9% vs*.* 18.7%, *P* < 0.001) (Fig. 4A). When transplantation was not treated as censored event, high transcription level of *ECM1* still had a worse 5-year OS (low vs*.* high, 71.1% vs*.* 56.8%, *P* = 0.038) (Fig. 4B). Six factors with *P* < 0.1, including *ECM1* transcription level (high vs*.* low), age (≥ vs*.* < 35 years old), white blood cell (WBC, ≥ vs*.* < 30 × 10^9^/L), *BCR::ABL1* (positive *vs.* negative), *IKZF1* deletion (positive *vs.* negative), and MFC-MRD after induction (positive *vs.* negative), were enrolled in the multivariate analysis (transplantation as censored event). Only high *ECM1* transcription level, high WBC count, and positive MFC-MRD after induction were independent risk factors for OS (Supplementary Table 6). When transplantation was not considered as censored event, a total of four factors, including *ECM1* transcription level, age, *BCR::ABL1*, and transplantation (yes *vs.* no), were enrolled in the multivariate analysis. Only *BCR::ABL1* was an independent risk factor for OS, while receiving transplantation was an independent protective factor (Supplementary Table7).

We further validated the clinical significance of *ECM1* in patients by a B cell ALL dataset GSE34861 from the GEO database, with a total of 191 patients. Samples in this database were obtained from bone marrow and peripheral blood. For greater comparability with our samples, we only enrolled 91 patients with samples obtained from bone marrow. The median transcription level of *ECM1* in these patients was 14.58 (9.22–15.57). According to the median transcription level, there were 45 cases in the low transcription level group and 46 in the high. The comparison of clinical information between the two groups was basically consistent with our patients (Supplementary Table 8). Pro-B and Pre-B were still mainly seen in the low transcription level group. *BCR::ABL1* mostly occurred in the high transcription level group, while *KMT2A*-r and *TCF3::PBX1* were still only found in the low transcription level group. A total of 74 patients were enrolled in the OS analysis (1 patient without follow-up information and 16 patients with *KMT2A*-r or *TCF3::PBX1* were excluded). Transplantation was not treated as censored event due to the lack of transplantation information in the dataset. Similar to Fig. 4B, patients with high *ECM1* transcription level still had a poor prognosis (37.4% vs*.* 10.6%, *P* = 0.059) (Supplementary Fig. 2).

**Fig. 5:**
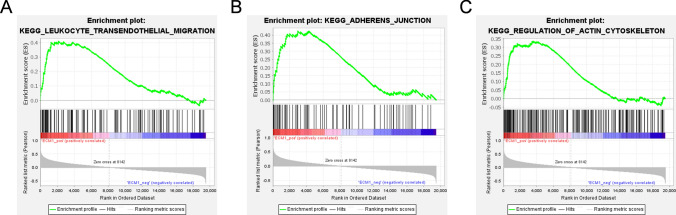
KEGG analysis of B cell ALL patients with high *ECM1* transcription level. **A** Leukocyte transendothelial migration, **B** Adherens junction, **C** Regulation of actin cytoskeleton

We further explored the potential mechanism by which high *ECM1* transcription levels affected the prognosis of B cell ALL. KEGG enrichment analysis of differentially expressed genes was performed in B cell ALL patients with high *ECM1* transcription level. We found 3 pathways with the highest enrichment score were involved in the regulation of cell migration, including leukocyte transendothelial migration, adherens junction and regulation of actin cytoskeleton (Fig. 5). These results suggested that *ECM1* may affect the invasion and metastasis of tumor cells. In the leukocyte transendothelial migration pathway, the genes that contribute primarily to enrichment score were *CD99*, *ACTN1*, and *CTNND1* (Supplementary Table 9).

## Discussion

In this study, we found that *ECM1* was highly transcribed in B cell ALL patients, which indicated *ECM1* was a potential biomarker for B cell ALL. Under the physiological conditions, *ECM1* participated in the formation of extracellular matrix, cell adhesion, signal transduction, and tissue differentiation and maturation [16]. Therefore, the abnormality of this gene would cause complex pathological changes. It has been reported that *ECM1* was over-expressed in many solid tumors, such as breast cancer, thyroid cancer, cholangiocarcinoma, lung cancer, bladder cancer, ovarian cancer, colorectal cancer, and was related to chemo-resistance, tumor invasion, metastasis and poor prognosis [17–26]. Other studies also found that *ECM1* was down-regulated in some tumors, such as esophageal cancer and prostate cancer, as a tumor suppressor [27, 28]. The above research suggests that *ECM1* may have distinguish mechanisms in different cancers. In recent years, it has been found that mutated p53 protein can cooperate with Notch1 to promote *ECM1* expression by relieving the promoter inhibition of *ECM1*, leading to T-ALL [29]. However, *ECM1* has not yet been reported in B cell ALL. In AML, *ECM1* was transcribed at a level comparable to that of the donor, which indicated a specific role of *ECM1* in ALL. ROC analysis indicated a good diagnostic performance of *ECM1* in B cell ALL, and in combination with *WT1* could further improve the sensitivity. These results indicated that *ECM1* could serve as a good auxiliary diagnostic biomarker for B cell ALL.

Interestingly, we found that *ECM1* showed significantly different transcription level in different genetic background, further confirming the heterogeneity of B cell ALL with different driver events. The transcription level of *ECM1* in *BCR::ABL1* positive patients was higher than that in *BCR::ABL1* negative, and the highest level was found in *BCR::ABL1* and *IKZF1* deletion double-positive patients. However, in *BCR::ABL1* negative subjects, *IKZF1* deletion had no significant correlation with *ECM1* transcription level. Diagnostic test evaluation suggested that the sensitivity and specificity of *ECM1* in the diagnosis of B cell ALL in patients with *BCR::ABL1* reached 100%. However, due to the sample size of healthy donors, the diagnostic performance of *ECM1* for *BCR::ABL1* subjects and whether there is a synergistic effect with *IKZF1* still need to be further explored. However, in patients with *KMT2A*-r and *TCF3::PBX1* rearrangement, the transcription level of *ECM1* decreased significantly, although still higher than the donors. Transcription level in *ETV6::RUNX1* patients was comparable to those in *BCR::ABL1* negative ones. Because most of the patients we enrolled were adults, the number of *ETV6::RUNX1* cases was too small to draw a solid conclusion. A larger sample size is needed to explore the *bona fide* transcription level in these patients.

Detecting MRD is an important disease course monitoring and risk stratification method in the clinical treatment of B cell ALL, with the advantage of being applicable to over 90% of patients [6]. At present, MRD detection is mainly performed in clinical practice by combining techniques such as flow cytometry and PCR. Stow et al*.* [30] showed that even if the MRD detected by flow cytometry was less than 0.01%, some patients still had a higher risk of relapse. Therefore, identify the deeper MRD level is the hot spot for MRD monitoring. Although the second-generation sequencing technology has a higher sensitivity (at least 10^–6^) than the aforementioned detection methods, further clinical application is limited due to its high cost. Overall, qRT-PCR is currently one of the most cost-effective and sensitive (over 10^–5^) MRD monitoring methods. *IGH* rearrangement is a widely used target in qRT-PCR MRD monitoring in B cell ALL patients. However, only approximately 50–75% of B cell ALL patients have typical *IGH* rearrangement [31].

Therefore, development of new molecular markers based on qRT-PCR MRD detection methods is crucial for assisting clinical course monitoring and prognosis prediction. We found that the dynamic transcription level of *ECM1* was consistent with the clinical course of B cell ALL. Compared with other MRD detection techniques, *ECM1* transcription level detection was not inferior than morphology, flow cytometry, and quantitative detection of other genes. These results suggest that *ECM1* is a good MRD monitoring indicator. Someone, with low transcription level of *ECM1* need to be evaluated with other techniques, especially in ones with *KMT2A*-r or *TCF3::PBX1*. Particularly, like the current MRD markers in clinical practice, the transcription level of *ECM1* did not indicate the status of extramedullary relapse well because the corresponding bone marrow was not infiltrated.

Finally, we found that in patients with transplantation as censored event, high *ECM1* transcription level had poor prognosis and was an independent risk factor for OS. The poor outcome may result from *ECM1* properties of promoting invasion and metastasis. *CD99* contributed mainly to the cell migration. Recent study reported that *CD99* also showed poor prognosis in B cell ALL [32]. Surprisingly, the transcription level of *CD99* among different subgroups in B cell ALL was quite similar to that of *ECM1*. Patients with *BCR::ABL1* had high *CD99* transcription level and ones with *KMT2A*-r and *TCF3::PBX1* had the lowest. Interestingly, patients with *CRLF2* rearrangements, one of the types of Ph-like ALL, showed highest *CD99* transcription level. These results indicated a closely relationship between *BCR::ABL1* signature and *CD99* transcription. *CD99* was a target of *MMP9* [33], a gene reducing cell migration. Reports also demonstrated that *ECM1* could activate or inhibit the activity of *MMP9* in different cancers and genodermatosis [21, 34, 35]. Collectively, *ECM1* may interact with *MMP9* and *CD99* directly or indirectly to promote cell migration in B cell ALL, especially ones with *BCR::ABL1*.

However, when considering transplantation, high transcription level of *ECM1* was no longer an independent risk factor. The poor prognosis caused by high transcription level of *ECM1* could be improved by transplantation. Therefore, patients with high transcription level of *ECM1* should be considered as high risk, and should be closely monitored and given a transplantation as much as possible.

In summary, we have identified *ECM1* as a new biomarker for B cell ALL. Due to the correlation between its transcription level and genetic background, the high transcription level of *ECM1* is more likely to be a secondary hit in the development of B cell ALL rather than a driving event [36], especially in patients with *BCR::ABL1* and *IKZF1* deletion. The effects of *BCR::ABL1* and *IKZF1* deletion on the biological function of *ECM1* still need further exploration. Further study of the molecular mechanisms of *ECM1* in different genetic backgrounds may provide a more comprehensive understanding of B cell ALL and promote personalized diagnosis and treatment.

## Supplementary Information

Below is the link to the electronic supplementary material.

Supplementary file1 (JPG 171 kb)

Supplementary file2 (JPG 97 kb)

Supplementary file1 (DOC 108 kb)

## Data Availability

Not applicable.

## Abbreviations

ALL: Acute lymphoblastic leukemia
CR: Complete remission
*ECM1*: Extracellular matrix protein 1
*KMT2A*-r: *KMT2A* rearrangement
MFC: Multi-parameter flow cytometry
MRD: Minimal residual disease
OS: Overall survival
qRT-PCR: Quantitative real-time polymerase chain reaction
ROC: Receiver operator characteristic

## References

[1] Iacobucci I, Mullighan CG. Genetic basis of acute Lymphoblastic Leukemia. J Clin Oncol. 2017;35(9):975–83. 28297628 10.1200/JCO.2016.70.7836 PMC5455679

[2] Tran TH, Langlois S, Meloche C, et al. Whole-transcriptome analysis in acute lymphoblastic leukemia: a report from the DFCI All Consortium Protocol 16–001. Blood Adv. 2022;6(4):1329–41. 34933343 10.1182/bloodadvances.2021005634 PMC8864659

[3] Gu Z, Churchman ML, Roberts KG, et al. PAX5-driven subtypes of B-progenitor acute lymphoblastic leukemia. Nat Genet. 2019;51(2):296–307. 30643249 10.1038/s41588-018-0315-5 PMC6525306

[4] Studd JB, Cornish AJ, Hoang PH, Law P, Kinnersley B, Houlston R. Cancer drivers and clonal dynamics in acute lymphoblastic leukaemia subtypes. Blood Cancer J. 2021;11(11):177. 34753926 10.1038/s41408-021-00570-9 PMC8578656

[5] Wu S, Liu L, Chu X, et al. Dynamic change of variant allele frequency reveals disease status, clonal evolution and survival in pediatric relapsed B-cell acute lymphoblastic leukaemia. Clin Transl Med. 2022;12(5):e892. 35605061 10.1002/ctm2.892 PMC9126496

[6] Moorman AV. New and emerging prognostic and predictive genetic biomarkers in B-cell precursor acute lymphoblastic leukemia. Haematologica. 2016;101(4):407–16. 27033238 10.3324/haematol.2015.141101 PMC5004393

[7] Yang M, Vesterlund M, Siavelis I, et al. Proteogenomics and Hi-C reveal transcriptional dysregulation in high hyperdiploid childhood acute lymphoblastic leukemia. Nat Commun. 2019;10(1):1519. 30944321 10.1038/s41467-019-09469-3 PMC6447538

[8] Mullighan CG. How advanced are we in targeting novel subtypes of all? Best Pract Res Clin Haematol. 2019;32(4):101095. 31779973 10.1016/j.beha.2019.101095 PMC6927537

[9] Aldoss I, Afkhami M, Yang D, et al. High response rates and transition to transplant after novel targeted and cellular therapies in adults with relapsed/refractory acute lymphoblastic leukemia with Philadelphia-like fusions. Am J Hematol. 2023;98(6):848–56. 36880203 10.1002/ajh.26908 PMC13743007

[10] Lilljebjörn H, Henningsson R, Hyrenius-Wittsten A, et al. Identification of ETV6::RUNX1-like and DUX4-rearranged subtypes in paediatric B-cell precursor acute lymphoblastic leukaemia. Nat Commun. 2016;7:11790. 27265895 10.1038/ncomms11790 PMC4897744

[11] Wang Q, Cai WZ, Wang QR, et al. Integrative genomic and transcriptomic profiling reveals distinct molecular subsets in adult mixed phenotype acute leukemia. Am J Hematol. 2023;98(1):66–78. 36219502 10.1002/ajh.26758

[12] Zhao XS, Yan CH, Liu DH, et al. Combined use of WT1 and flow cytometry monitoring can promote sensitivity of predicting relapse after allogeneic HSCT without affecting specificity. Ann Hematol. 2013;92(8):1111–9. 23680867 10.1007/s00277-013-1733-1

[13] Lai YY, Huang XJ, Li J, et al. Standardized fluorescence in situ hybridization testing based on an appropriate panel of probes more effectively identifies common cytogenetic abnormalities in myelodysplastic syndromes than conventional cytogenetic analysis: a multicenter prospective study of 2302 patients in China. Leuk Res. 2015;39(5):530–5. 25823643 10.1016/j.leukres.2015.02.005

[14] Ruan GR, Qin YZ, Chen SS, et al. Abnormal expression of the programmed cell death 5 gene in acute and chronic myeloid leukemia. Leuk Res. 2006;30(9):1159–65. 16507320 10.1016/j.leukres.2005.12.028

[15] Schittek B, Sinnberg T. Biological functions of casein kinase 1 isoforms and putative roles in tumorigenesis. Mol Cancer. 2014;13:231. 25306547 10.1186/1476-4598-13-231 PMC4201705

[16] Dai Z, Cai L, Chen Y, et al. Brusatol inhibits proliferation and invasion of glioblastoma by Down-Regulating the expression of ECM1. Front Pharmacol. 2021;12:775680. 34970146 10.3389/fphar.2021.775680 PMC8713816

[17] Steinhaeuser SS, Morera E, Budkova Z, et al. ECM1 secreted by HER2-overexpressing breast cancer cells promotes formation of a vascular niche accelerating cancer cell migration and invasion. Lab Invest. 2020;100(7):928–44. 32203150 10.1038/s41374-020-0415-6

[18] Bergamaschi A, Tagliabue E, Sørlie T, et al. Extracellular matrix signature identifies breast cancer subgroups with different clinical outcome. J Pathol. 2008;214(3):357–67. 18044827 10.1002/path.2278

[19] Kebebew E, Peng M, Reiff E, Duh QY, Clark OH, McMillan A. ECM1 and TMPRSS4 are diagnostic markers of malignant thyroid neoplasms and improve the accuracy of fine needle aspiration biopsy. Ann Surg. 2005;242(3):353–61. 16135921 10.1097/01.sla.0000179623.87329.6b PMC1357743

[20] Jeong S, Lee SG, Kim H, et al. Simultaneous expression of long non-coding RNA FAL1 and extracellular matrix protein 1 defines tumour behaviour in young patients with papillary thyroid cancer. Cancers (Basel). 2021;13(13):3223. 34203279 10.3390/cancers13133223 PMC8268647

[21] Xiong GP, Zhang JX, Gu SP, Wu YB, Liu JF. Overexpression of ECM1 contributes to migration and invasion in cholangiocarcinoma cell. Neoplasma. 2012;59(4):409–15. 22489696 10.4149/neo_2012_053

[22] Yin H, Jiang Z, Feng X, Ji Z, Jin W. Identification of Sca-1(+)Abcg1(+) bronchioalveolar epithelial cells as the origin of lung adenocarcinoma in Gprc5a-knockout mouse model through the interaction between lung progenitor AT2 and Lgr5 cells. Oncogene. 2020;39(18):3754–73. 32157214 10.1038/s41388-020-1251-2 PMC7190569

[23] Wang J, Guo M, Zhou X, et al. Angiogenesis related gene expression significantly associated with the prognostic role of an urothelial bladder carcinoma. Transl Androl Urol. 2020;9(5):2200–10. 33209684 10.21037/tau-20-1291 PMC7658114

[24] Chen H, Jia WD, Li JS, et al. Extracellular matrix protein 1, a novel prognostic factor, is associated with metastatic potential of hepatocellular carcinoma. Med Oncol. 2011;28(Suppl 1):S318-325. 21128013 10.1007/s12032-010-9763-1

[25] Lv C, Ren C, Yu Y, et al. Wentilactone a reverses the NF-κB/ECM1 signaling-induced cisplatin resistance through inhibition of IKK/IκB in ovarian cancer cells. Nutrients. 2022;14(18):3790. 36145166 10.3390/nu14183790 PMC9504226

[26] Long S, Wang J, Weng F, et al. ECM1 regulates the resistance of colorectal cancer to 5-FU treatment by modulating apoptotic cell death and epithelial-mesenchymal transition induction. Front Pharmacol. 2022;13:1005915. 36408224 10.3389/fphar.2022.1005915 PMC9666402

[27] Yu VZ, Ko JMY, Ning L, Dai W, Law S, Lung ML. Endoplasmic reticulum-localized ECM1b suppresses tumor growth and regulates MYC and MTORC1 through modulating MTORC2 activation in esophageal squamous cell carcinoma. Cancer Lett. 2019;461:56–64. 31319137 10.1016/j.canlet.2019.07.005

[28] Al Shareef Z, Kardooni H, Murillo-Garzón V, et al. Protective effect of stromal Dickkopf-3 in prostate cancer: opposing roles for TGFBI and ECM-1. Oncogene. 2018;37(39):5305–24. 29858602 10.1038/s41388-018-0294-0 PMC6160402

[29] Zhang J, Sun W, Kong X, et al. Mutant p53 antagonizes p63/p73-mediated tumor suppression via Notch1. Proc Natl Acad Sci U S A. 2019;116(48):24259–67. 31712410 10.1073/pnas.1913919116 PMC6883818

[30] Stow P, Key L, Chen X, et al. Clinical significance of low levels of minimal residual disease at the end of remission induction therapy in childhood acute lymphoblastic leukemia. Blood. 2010;115(23):4657–63. 20304809 10.1182/blood-2009-11-253435 PMC2890183

[31] Logan AC, Vashi N, Faham M, et al. Immunoglobulin and T cell receptor gene high-throughput sequencing quantifies minimal residual disease in acute lymphoblastic leukemia and predicts post-transplantation relapse and survival. Biol Blood Marrow Transplant. 2014;20(9):1307–13. 24769317 10.1016/j.bbmt.2014.04.018 PMC5259557

[32] Chen D, Camponeschi A, Wu Q, et al. CD99 expression is strongly associated with clinical outcome in children with B-cell precursor acute lymphoblastic leukaemia. Br J Haematol. 2019;184(3):418–23. 30484860 10.1111/bjh.15683

[33] Aguilera-Montilla N, Bailón E, Uceda-Castro R, et al. MMP-9 affects gene expression in chronic lymphocytic leukemia revealing CD99 as an MMP-9 target and a novel partner in malignant cell migration/arrest. Oncogene. 2019;38(23):4605–19. 30760844 10.1038/s41388-019-0744-3

[34] Zhang Y, Wang W, Zhou H, Cui Y. Urinary eubacterium sp. CAG:581 promotes non-muscle invasive bladder cancer (NMIBC) development through the ECM1/MMP9 pathway. Cancers (Basel). 2023;15(3):809. 36765767 10.3390/cancers15030809 PMC9913387

[35] Fujimoto N, Terlizzi J, Aho S, et al. Extracellular matrix protein 1 inhibits the activity of matrix metalloproteinase 9 through high-affinity protein/protein interactions. Exp Dermatol. 2006;15(4):300–7. 16512877 10.1111/j.0906-6705.2006.00409.x

[36] Irving JA, Enshaei A, Parker CA, et al. Integration of genetic and clinical risk factors improves prognostication in relapsed childhood B-cell precursor acute lymphoblastic leukemia. Blood. 2016;128(7):911–22. 27229005 10.1182/blood-2016-03-704973 PMC5026463

