# Supplementary figures and images for: Extracellular matrix protein 1 (ECM1) is a potential biomarker in B cell acute lymphoblastic leukemia
Source: Clin Exp Med. 2024 Mar 28;24(1):56. doi: 10.1007/s10238-023-01255-2 (PMC10978711; doi:10.1007/s10238-023-01255-2)

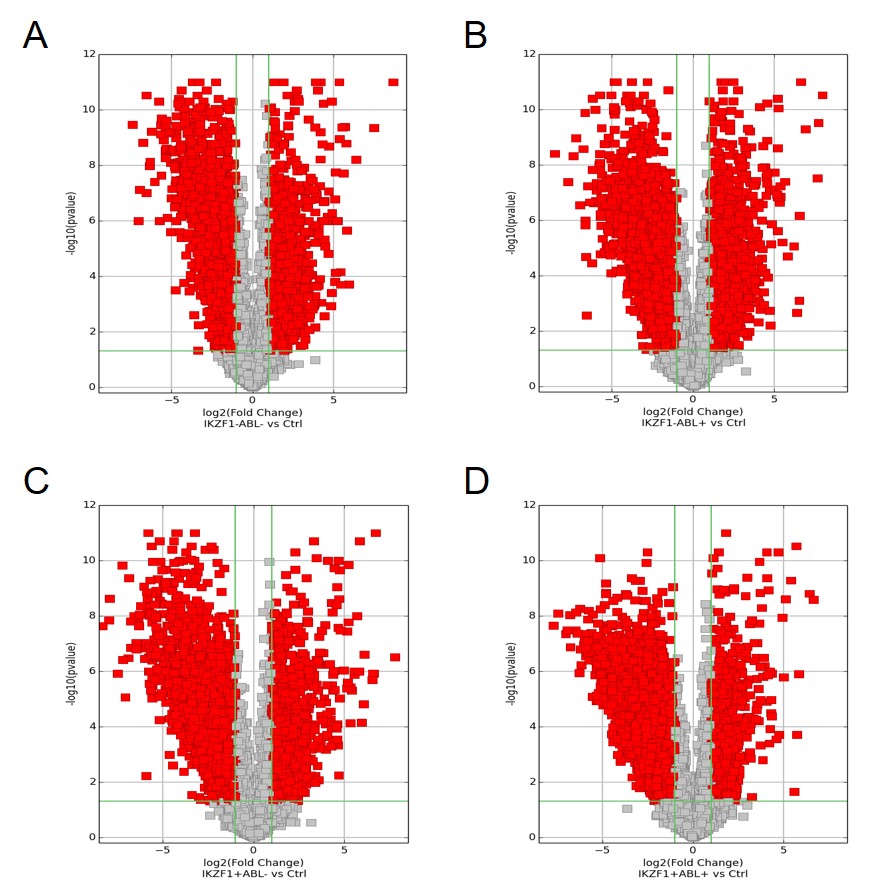

Supplement: Supplementary file 1 — Supplementary file1 (JPG 171 kb) [file 10238_2023_1255_MOESM1_ESM.jpg]

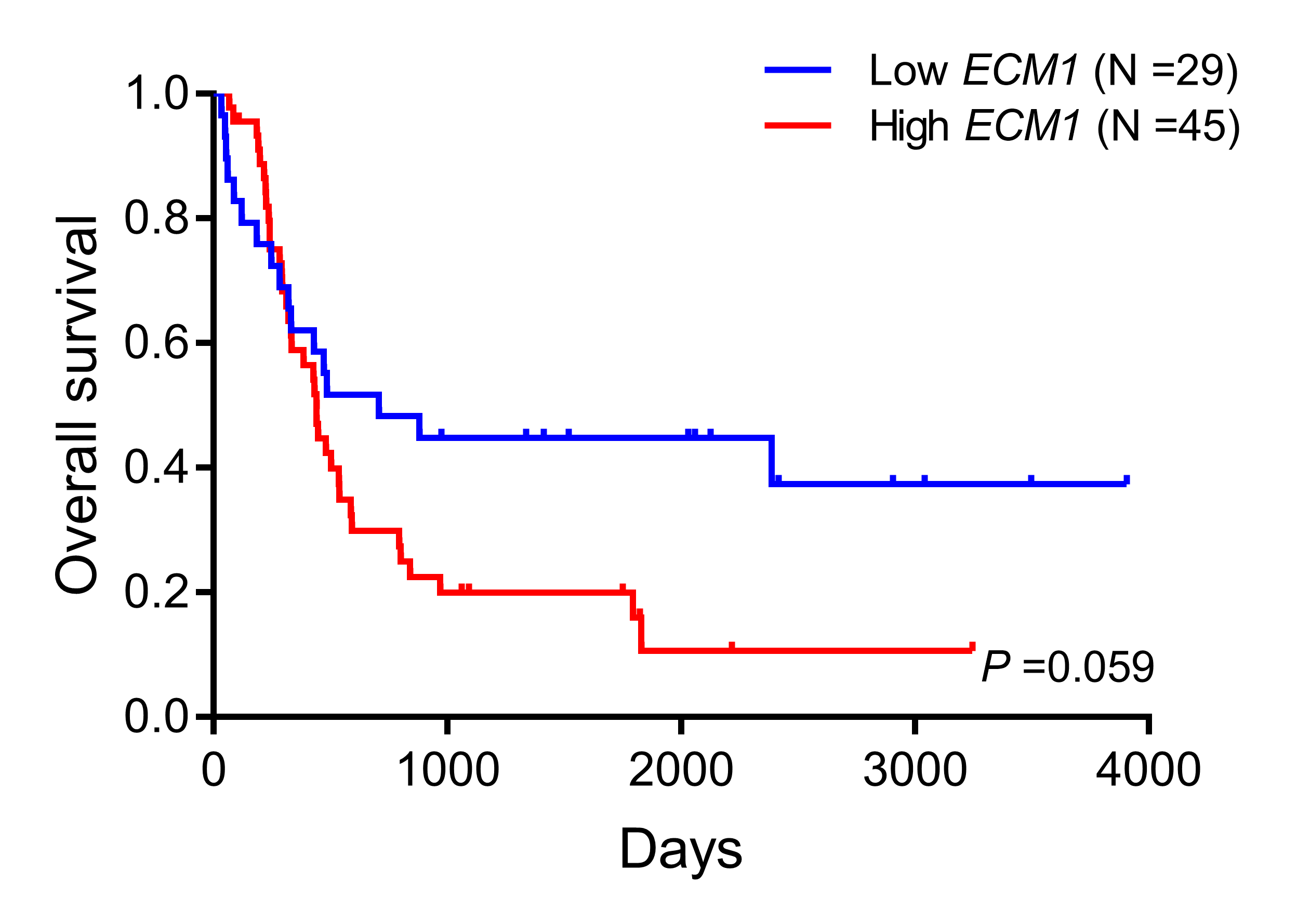

Supplement: Supplementary file 2 — Supplementary file2 (JPG 97 kb) [file 10238_2023_1255_MOESM2_ESM.jpg]
