# Supplementary material for: Extracellular matrix protein 1 (ECM1) is a potential biomarker in B cell acute lymphoblastic leukemia
Source: Clin Exp Med. 2024 Mar 28;24(1):56. doi: 10.1007/s10238-023-01255-2 (PMC10978711; doi:10.1007/s10238-023-01255-2)
Supplement: Supplementary file 3 — Supplementary file1 (DOC 108 kb) [file 10238_2023_1255_MOESM3_ESM.doc]

**Supplementary Materials**

**Figure**

**Supplementary Figure 1. Volcano plot of dysregulated genes in B-cell ALL.** Red squares indicated the significant genes with fold change ≥2 (right side) or ≤0.5 (left side). (A) Double-negative group; (B) *BCR::ABL1*-positive group; (C) *IKZF1*-positive group; (D) Double-positive group. Ctrl: normal donor control.

**Supplementary Figure 2. Prognostic analysis of *ECM1* in B-cell ALL patients from GSE34861.**

**Table**

**Supplementary Table 1. Patient characteristics for gene expression microarray**

| **Variables** | **B-cell ALL (N=30)** |
| --- | --- |
| **Sex, *N* (%)** |  |
| Male | 19 (63) |
| Female | 11 (37) |
| **Age, y** |  |
| Median (range) | 33 (14~79) |
| **WBC, ×10^9^/L** |  |
| Median (range) | 27.37 (1.10~416.52) |
| **Hemoglobin, g/L** |  |
| Median (range) | 99 (44 ~158) |
| **Platelets, ×10^9^/L** |  |
| Median (range) | 77 (4 ~265) |
| **Blast, %** |  |
| Median (range) | 89 (21~99) |
| **Immunophenotype, *N* (%)** |  |
| Common-B | 22 (73) |
| Pro-B | 4 (13) |
| Pre-B | 4 (13) |
| **Cytogenetics, *N* (%)** |  |
| t(9;22)(q34;q11) | 11 (37) |
| t(4;11)(q21;q23) | 2 (7) |
| t(1;19)(q23;p13) | 1 (3) |
| Normal karyotype | 8 (27) |
| Other karyotype | 4 (13) |
| Unknown | 4 (13) |
| **Molecular aberrations,** ***N* (%)** |  |
| *BCR::ABL1* | 14 (47) |
| *IKZF1* deletion | 14 (47) |
| *KMT2A*-r | 5 (17) |
| *TCF3::PBX1* | 1 (3) |
| *ETV6::RUNX1* | 0 |

**Supplementary Table 2. Dysregulated genes in B-cell ALL subgroups**

| **Subgroup** | **Upregulated genes** | **Downregulated genes** |
| --- | --- | --- |
| *BCR::ABL1*(-)/*IKZF1*(-) *vs.* donor | 2024 | 2451 |
| *BCR::ABL1*(+)/*IKZF1*(-) *vs.* donor | 2724 | 3542 |
| *BCR::ABL1*(-)/*IKZF1*(+) *vs.* donor | 1955 | 3553 |
| *BCR::ABL1*(+)/*IKZF1*(+) *vs.* donor | 1486 | 4195 |

**Supplementary Table 3. Fold change of *ECM1* transcription level in B-cell ALL subgroups**

| **Subgroup** | **NM_004425** | ***P*-value** | **NM_022664** | ***P*-value** |
| --- | --- | --- | --- | --- |
| *BCR::ABL1*(-)/*IKZF1*(-) *vs.* donor | 29 | 4.03×10^-4^ | 42 | 6.98×10^-5^ |
| *BCR::ABL1*(+)/*IKZF1*(-) *vs.* donor | 123 | 5.00×10^-10^ | 211 | 3.00×10^-10^ |
| *BCR::ABL1*(-)/*IKZF1*(+) *vs.* donor | 40 | 3.76×10^-6^ | 94 | 1.19×10^-6^ |
| *BCR::ABL1*(+)/*IKZF1*(+) *vs.* donor | 34 | 2.46×10^-9^ | 85 | 1.56×10^-9^ |

**Supplementary Table 4. Patient characteristics for 267 B-cell ALL in the validation group**

| **Variables** | **B-cell ALL (N=267)** |
| --- | --- |
| **Sex, *N* (%)** |  |
| Male | 145 (54) |
| Female | 122 (46) |
| **Age, y** |  |
| Median (range) | 32 (1~71) |
| **WBC, ×10^9^/L** |  |
| Median (range) | 15.46 (0.78~464.39) |
| **Hemoglobin, g/L** |  |
| Median (range) | 91 (13 ~172) |
| **Platelets, ×10^9^/L** |  |
| Median (range) | 52 (3 ~377) |
| **Immunophenotype, *N* (%)** |  |
| Common-B | 198 (74) |
| Pro-B | 38 (14) |
| Pre-B | 26 (10) |
| Unknown | 5 (2) |
| **Molecular aberrations,** ***N* (%)** |  |
| *BCR::ABL1* | 187 (70) |
| *IKZF1* deletion | 119 (45) |
| *KMT2A*-r | 13 (5) |
| *TCF3::PBX1* | 13 (5) |
| *ETV6::RUNX1* | 7 (3) |

**Supplementary Table 5. Diagnostic performance of *ECM1* and *WT1* in B-cell ALL***

| ***ECM1*** | ***WT1*** | |
| --- | --- | --- |
|  | + | - |
| + | 145 | 80 |
| - | 29 | 11 |

*: Threshold of *ECM1* transcription level was 10.28%, and 0.60% for *WT1*.

**Supplementary Table 6.** **Multivariate analysis (transplantation as censored event)**

| **Factors** | **HR（95% CI）** | ***P*-value** |
| --- | --- | --- |
| *ECM1* transcription level (high *vs.* low) | 5.77 (1.75~19.06) | 0.004 |
| WBC (≥ *vs.* <30×10^9^/L) | 3.51 (1.21~10.17) | 0.021 |
| MFC-MRD after induction (+ *vs*. -) | 3.83 (1.38~10.64) | 0.010 |

**Supplementary Table 7. Multivariate analysis (transplantation not as censored event)**

| **Factors** | **HR（95% CI）** | ***P*-value** |
| --- | --- | --- |
| *BCR::ABL1* (+ *vs.* -) | 2.22 (1.27~3.89) | 0.005 |
| Transplantation (yes *vs.* no) | 0.22 (0.12~0.38) | <0.001 |

**Supplementary Table 8. Characteristics of 91 B-cell ALL patients from GSE34861**

| **Variables** | ***ECM1* high (N =46)** | ***ECM1* low (N =45)** | ***P*-value** |
| --- | --- | --- | --- |
| **Sex, *N* (%)** |  |  | 0.926 |
| Male | 26 (57) | 25 (56) |  |
| Female | 20 (43) | 20 (44) |  |
| **Age, y** |  |  |  |
| Median (range) | 40 (19~59) | 38 (19~62) | 0.268 |
| **WBC, ×10^9^/L** |  |  |  |
| Median (range) | 31.9 (0.8~438.0) | 26.4 (1.0~295.1) | 0.971 |
| **Immunophenotype, *N* (%)** |  |  | **<0.001** |
| Common-B | 42 (91) | 21 (47) |  |
| Pro-B | 0 | 14 (31) |  |
| Pre-B | 2 (4) | 7 (16) |  |
| unknown | 2 (4) | 3 (7) |  |
| **Molecular/Cytogenetics, *N* (%)** |  |  |  |
| *BCR::ABL1* | 21 (46) | 12 (27) | 0.060 |
| *KMT2A*-r | 0 | 11 (24) | **<0.001** |
| *TCF3::PBX1* | 0 | 5 (11) | **0.026** |
| Undefined | 25 (54) | 16 (36) | 0.072 |

**Supplementary Table 9. Top 10 core enrichment genes in the leukocyte transendothelial migration pathway**

| **Symbol** | **Rank in gene list** | **Rank metric score** | **Running Enrichment Score** |
| --- | --- | --- | --- |
| *CD99* | 7 | 0.676 | 0.0328 |
| *ACTN1* | 31 | 0.6 | 0.061 |
| *CTNND1* | 77 | 0.528 | 0.0846 |
| *ARHGAP5* | 212 | 0.443 | 0.0994 |
| *PTK2B* | 255 | 0.423 | 0.118 |
| *ITGB1* | 325 | 0.401 | 0.1341 |
| *RAPGEF3* | 344 | 0.394 | 0.1525 |
| *GNAI1* | 363 | 0.388 | 0.1707 |
| *PIK3R5* | 364 | 0.388 | 0.1897 |
| *NCF2* | 516 | 0.358 | 0.1994 |
